# Exploring the relationship between mental health-related problems and undergraduate student dropout: A case study within a civil engineering program

**DOI:** 10.1016/j.heliyon.2022.e09504

**Published:** 2022-05-25

**Authors:** A.A. Del Savio, K. Galantini, A. Pachas

**Affiliations:** a Civil Engineering Program, Universidad de Lima, Lima, Peru; b Architecture Program, Universidad de Lima, Lima, Peru

**Keywords:** Undergraduate dropout, Dropout rates, Mental health, COVID-19

## Abstract

Dropout has always been a concerning issue within higher education. This research explores the relationship between mental health-related problems and undergraduate students’ dropout rates amid the COVID-19 pandemic and within a civil engineering program. The study is quantitative descriptive, with a non-experimental and longitudinal design. Results show that the dropout rates of civil engineering students from a private university have increased, comparing before and after the COVID-19 pandemic, primarily due to mental health-related problems. Three strong tendencies within these rates have been detected, directly related to the COVID-19 pandemic. It was concluded that undergraduate students’ mental health had been undermined due to the pandemics, which has had an immediate impact on the increase of dropout rates.

## 1 Introduction

Since 2020, one of the most impacted sectors due to the disruptive effects of the COVID-19 has been education (UNESCO, 2021; Schleicher, 2020; Tadesse and Muluye, 2020). Within the higher education context, the transition to online learning environments, due to confinement measures, became a sudden and inevitable obligation for the entire academic community. The digital transformation was forced within a short period, leaving no gap for a progressive adaptation process (Warfvinge et al., 2021).

Nevertheless, this transformation has brought some significant improvements for the training processes, such as flexibility, interactivity, personalization of the teaching-learning and assessment processes (Amer 2007), and easier access and share of information (Wang 2008). It also led to dares, such as limited access to the internet (Fruehwirth et al., 2021), general work overload (Padrón et al., 2021), faculty members' skills to deal with technology (Morales-Rodríguez, 2021), students’ financial issues to acquire ICT materials, infrastructural deficiency, restricted laboratory and practical courses, controversial online assessment and evaluation (Hossain et al., 2021), “multitasking” (Alvarez-Risco et al., 2021), among others.

Furthermore, within higher education, literature reports educational consequences of the COVID-19, such as decreased participation in higher education programs, delays in studies because of financial problems, cancellation of practical training and work placements, failure of continuation of internships, postponement of research projects, technological equipment dependency, among others (de Boer, 2021; Warps and van den Broek, 2020; Adedoyin and Soykan, 2020). Additionally, according to a survey carried out by UNESCO (2020), the main problems faced by Ibero-American students due to COVID-19 were access to the internet, economic issues, keeping a regular schedule, and lack of ICT equipment.

One of the main challenges of online education is student dropout. Online programs have shown higher dropout rates than face-to-face programs (Dash et al., 2021; Grau-Valldosera and Minguillón, 2014). There are many conceptions for the term “student dropout” (Xavier and Meneses, 2020), but all of them are often related to the variable time (Grau-Valldosera and Minguillón, 2014). It can be defined as the withdrawal from courses for some time before their completion (Lim, 2016). Student dropout has been reported to negatively impact students’ self-esteem and psychological, emotional, and social well-being (Ayala and Manzano, 2018), ultimately undermining learning.

In a similar scenario, mental health has been a significant issue across the COVID-19 pandemic due to the uncertainty faced (Rettie and Daniels, 2021). Among the most frequent mental health disorders reported, the ones related to negative emotions such as anxiety, depression, stress, and distress stand out (Browning et al., 2021; Fruehwirth et al., 2021; Hossain et al., 2021). The World Health Organization (WHO) exposes that dealing with telecommuting, home school, and lack of physical contact has demanded new adaptation skills for people to change their routines and reorganize their activities (Alhawsawi and Jawhar, 2021). Moreover, psychological impacts of the pandemic in higher education students include increased cases of anxiety (Wang et al., 2020), depression, eating disorders, alcohol and drug consumption (Kohls et al., 2020), stress (Van de Velde et al., 2020), among others.

Therefore, it can be observed that mental health disorders, which can be related to general dissatisfaction with the online training processes (Khawar et al., 2021; Fruehwirth et al., 2021; Browning et al., 2021), have a negative impact on students’ learning (Lipson and Eisenberg, 2017) since they inhibit the formation of memory and lead to a lack of motivation. Added to unsatisfactory academic outcomes because of what was previously addressed (Abdelrahman, 2020), these circumstances can lead to student dropout. This research shows a case study within a civil engineering program that compares students’ dropout rate before and after the COVID-19 pandemic, dropouts that respond to previously diagnosed mental health-related problems, from 2018 to 2021. Therefore, the question formulated is:

• What are the differences in the undergraduate dropout rate of civil engineering students from a private university before and after the COVID-19 pandemic?

To answer it, the undergraduate dropout rate of civil engineering students from a private university due to the COVID-19 pandemic will be determined, as well as this pandemic’s most common mental health-related problems associated to undergraduate dropouts. The dependent and independent variables will be addressed: student dropout and mental health.

### 1.1 Student dropout

There is no standard definition of undergraduate dropout (Grau-Valldosera and Minguillón, 2014; Lee and Choi, 2011). It can be defined as the act to abandon an educational institution or classroom; the interruption of studies forever, without completing the corresponding curriculum; or the inactivity for three consecutive semesters, ignoring whether the students will resume their activities (Viale Tudela, 2014). Due to this lack of consensus, Tinto (1989) recommends adopting the most appropriate concept based on the study. Therefore, dropout is defined as the voluntary interruption of studies before concluding them for a period of time (Lim, 2016; Grau-Valldosera and Minguillón, 2014).

First, the intention to dropout is higher in males than females (Hovdhaugen, 2009). Several factors have been associated with university students’ intention to dropout, such as a lousy learning environment, unsatisfactory teaching, or being employed (Hovdhaugen and Aamodt, 2009). There are also reported causes related to emotional and mental health, such as burnout symptoms, lack of social support (Mostert and Pienaar, 2020), low levels of intrinsic motivation (Rump et al., 2017), and lack of self-esteem (Cortes et al., 2014).

Dropout rates are significantly greater among students with mental health-related problems (Ishii et al., 2018). According to the National Alliance on Mental Illness (NAMI, 2012), in the USA, more than 60% of higher education students who dropout did not continue their studies because of mental health-related reasons. Similarly, in the UK, around 60% of students have considered dropping out due to mental health-related problems (Unite Students, 2016). In the case of Peru, two studies that relate dropouts and mental health-related issues within a medicine student population have been conducted. The first one, that analyzed dropout rates in 2010, concluded that 22% of the students had at least one mental health-related problem (León-Jimenez et al., 2012); while the second one, that examined these rates between 2006 and 2014, found that only 5.7% withdrew due to mental health-related reasons (Necochea Ocaña et al., 2017).

On the other hand, learning-related emotions such as optimism about being a student and a sense of enjoyment about learning are reported to make the most substantial contribution to the students’ intention to finish their studies (Ekornes, 2021). These are also related to academic persistence, satisfaction, and motivation to continue their studies (Lipson and Eisenberg, 2017). Besides, the resilience factor is more prevalent in females in relation to their academic performance; their ability not to be quickly frustrated is one of the reasons for better performance than men (Ayala and Manzano, 2018).

Additionally, students enrolled in a scientific degree have a 70% higher probability of leaving their studies than social sciences and humanities students (Arias and Dehon, 2013). A study in Ecuador, conducted between 2015 and 2016, revealed that engineering degrees have a higher percentage of student dropout and that 27.2% of the students have thought about dropping out are due to the lack of motivation (Sinchi and Gómez, 2018). Furthermore, a study in Colombia, conducted between 2012 and 2017, concluded that more than 75% of the engineering students who dropout experienced stress and frustration that could not be solved (Prieto Mendez, 2018).

Within an engineering program, Pocock (2012) classified students’ reasons for dropping out into two: academic or non-academic (Marra et al., 2012; Meyer and Marx, 2014). Negative interactions with peers and instructors were also identified (Litzler and Young, 2012). It is then necessary to improve social inclusion and emotional support to prevent dropouts (Ekornes, 2021).

First and second-year students have a greater probability of leaving their engineering studies than students of other professional fields (Hartman and Hartman, 2006; Litzler and Young, 2012). Older students, who are enrolled in further years of the curriculum, are more prone to enjoy the learning process since they present more positive emotions related to the acquisition of knowledge (Ekornes, 2021), thus are less likely to dropout.

During the COVID-19 pandemic, few studies have been developed regarding students’ dropouts from universities due to mental health-related reasons. (Chi et al., 2021) found an association between poor mental health and students' intention to leave, which the pandemic has exacerbated. However, the intention to leave the program does not necessarily become a dropout. In addition, there is a relationship between lower levels of depression and students’ intention to dropout (Teuber et al., 2021). This intention could be related to their dissatisfaction with the changes in the training model, high level of academic burnout, and emotional stress (López-Aguilar and Álvarez-Pérez, 2021).

### 1.2 Mental health

According to the WHO (2018), mental health is defined as a state of complete mental, physical and social wellbeing. Mental health is more than the absence of mental disorders; it is a state where individuals can deal with everyday life stress and work productively. In addition, mental health gives human beings the capacity to think, feel, and act to improve their ability to enjoy life and deal with its challenges (Government of Canada, 2006). Therefore, mental health protection should be considered a significant concern within universities.

Regarding mental health and higher education, a study in the USA at four universities showed that 40% of their students have at least one mental health-related problem (Lipson and Eisenberg, 2017). Similarly, 59% of the students at a Canadian university have moderate to high levels of depression, and 56.3% reported the same in anxiety levels (Suresh et al., 2021). In a Spanish university, 34.9% of the students evaluated had symptoms of depression; 39.6% symptoms of anxiety; and 28.8%, symptoms of stress (Blanco et al., 2021). Other research found depression in around 13% of the studied population of undergraduate students (Hossain et al., 2021). Women are more likely to develop depression, anxiety, distress, and stress symptoms than men (Ibrahim et al., 2013; Elmer et al., 2020). Finally, the WHO estimates that, among higher education, the average rate of depression is 1 in 20, that is, 5% of the general undergraduate and graduate population (WHO, 2012).

### 1.3 Transition to online learning

In 2020, due to the global health emergency and COVID-19, the transition to online learning environments has led to several challenges. Literature has reported that the pandemic has largely undermined undergraduate students’ mental health. Difficulties in distance learning, academic overload, interpersonal conflicts, and social isolation have enormously contributed to university students' increased anxiety, depression, stress, and irritability symptoms (Elmer et al., 2020; Fruehwirth et al., 2021; Padrón et al., 2021).

Because there are still stigmas regarding mental health, students have not asked for help to attain it (Vivanco-Vidal et al., 2020). Moreover, under-recognition of the symptoms of mental health-related problems has been reported in undergraduate students (Furnham et al., 2011). As a result, stress and anxiety, particularly in engineering students, have led to medical conditions (Capetillo et al., 2019) and a deterioration of overall wellbeing (Moss et al., 2021).

There are no studies that relate the students’ mental health deterioration due to the COVID-19 and the undergraduate dropout rates. There are reports about how these rates have increased during the pandemic (McKinsey and Company, 2020), but none delves into the reasons. On the other hand, literature connects mental health and the intention of dropping out (Rump et al., 2017; Vallerand et al., 1997), but not the actual action. Thus, this research intends to fill this gap by studying a particular case within a civil engineering program.

## 2 Materials and methods

### 2.1 Context

The study was carried out within the civil engineering program of a Peruvian private higher education institution*.* The program is committed to ensuring its students' well-being by balancing the physical, psychological, social, and emotional aspects of students’ lives (Mahatmya et al., 2018). Therefore, employment opportunities, health counselling, extracurricular socializing activities, sports, and lifestyle services, among other resources, are considered. One of these is the competency-based curriculum (Del Savio et al., 2021), as generic and specific competencies support the integral development of human beings based on achieving their learning potential to know, do, coexist, and be (Tobón, 2013). Thus, the program is focused on enhancing lifelong learning (Del Savio et al., 2021), which is related to higher levels of motivation and achievement (Martin, 2012) and, therefore, learning outcomes. This aligns with the institution's mission, which focuses on training leading and creative professionals committed to society’s welfare.

Regarding the schedules of the program's different subjects, approximately 50% of the lectures are imparted during morning hours, between 7.00 and 12.00. Then, 25% of the lectures are programmed during noon, between 12.00 and 19.00 h, and the 25% left are delivered at night, between 19.00 and 22.00 h. This flexibility is sought to enable students to start their internships in their early years of studies since they are expected to enter the professional field as soon as they graduate.

In this context, since 2020, due to the COVID-19 pandemic, the dropout rates within the mentioned program have significantly risen compared to those before the said year. Most of them are direct consequences of diagnosed mental health-related problems, such as anxiety, stress, and distress. As the university where this study was carried out is interested in continuous improvement, the information was shared with anonymous consent, keeping the students’ privacy. The Research Board from *Universidad de Lima* revised this research and provided approval.

### 2.2 Purpose of the study

This study shows a case study within a civil engineering program that compares students’ dropout rates before and after the COVID-19 pandemic. These dropouts respond to previously diagnosed mental health-related problems, such as anxiety, depression, and distress. Therefore, the study variables are student dropout, the dependent one, and mental health-related issues, the independent one.

About the research design, the scope is descriptive since the objective is to specify the characteristics of a phenomenon the dropout rates to determine tendencies in a study group. Also, the research is non-experimental. It was carried out without the deliberate manipulation of the study variables and longitudinal, as data was collected at different points to make deductions about the evolution of the research problem. Finally, the approach is quantitative (Hernández et al., 2014).

### 2.3 Population and sample

This case study was carried out within the civil engineering program at a private university. According to the curriculum, the population involved students enrolled in five different program years, ranging from 17 to 24 years. Therefore, the majority are at the peak range of the onset of mental health-related problems, since literature suggests that 75% of diagnosable mental health problems start before the age of 25 (Kessler and Wang, 2008). The sample matches the population, as the gathered data encompasses the total number of students of the program.

About the sample characteristics, the number of students enrolled in every academic semester is the most distinctive trait of the population, is as shown in Table 1. Since the civil engineering program has an updated curriculum, according to the industry needs, the general registration numbers, despite the COVID-19 pandemic, have still increased year by year. On the other hand, Table 1 presents the gender distribution within the sample. It can be affirmed that the female and male ratio is approximately 1:4, respectively. This is consistent with other engineering programs, as reported by literature (Meyer and Marx, 2014; Marra et al., 2012).

**Table 1:** Sample distribution according to the year of the curriculum and gender.

Academic semester	Number of students enrolled		
	Female (%)	Male (%)	Total (100%)
2018–1	65 (23.81%)	208 (76.19%)	273
2018–2	76 (25.33%)	224 (74.67%)	300
2019–1	95 (23.99%)	301 (76.01%)	396
2019–2	99 (23.57%)	321 (76.43%)	420
2020–1	105 (20.79%)	400 (79.21%)	505
2020–2	109 (20.26%)	429 (79.74%)	538
2021–1	133 (20.75%)	508 (79.25%)	641

### 2.4 Data collection techniques and instruments

About the timing, the data collection about undergraduate student dropouts’ cases was held from 2018 to 2021, which includes the pre-and post-pandemic period. The monitoring was carried out over four consecutive years, starting when students first transitioned from the one-year introductory general studies program to the civil engineering program, when they began with subjects specific to their degree. In each of the four addressed years, information corresponds to the two regular semesters held. The first one, semester 1, takes place from April to July, and the second, semester 2, from August to December.

Every student dropout case was recorded correctly, including the following information: full name, code, semester, motive, medical report, date, and type. Three issues were considered about the explanation for the dropout: mental health-related reasons, economic factors, and others. In the cases where the dropout corresponded to more than one of the mentioned, mental health-related reasons were prioritized due to the nature of this research. About the type, the dropout could be total and partial, meaning students withdraw from all the courses or some of them, respectively.

About data analysis, quantitative simple is used, as this research informs about descriptive statistics, according to the classification provided by Malmi et al. (2016) for engineering education research papers.

## 3 Results and discussion

The analysis of this case study results shows that the undergraduate dropout rates of a civil engineering program from a private university have significantly risen, comparing the data from before and after the COVID-19 pandemic. An increase in the dropout cases was expected, reaching the 2018–2020 lapse with 2020 and 2021, the years of the pandemic (McKinsey and Company, 2020; Azevedo et al., 2021).

**Figure 1:**
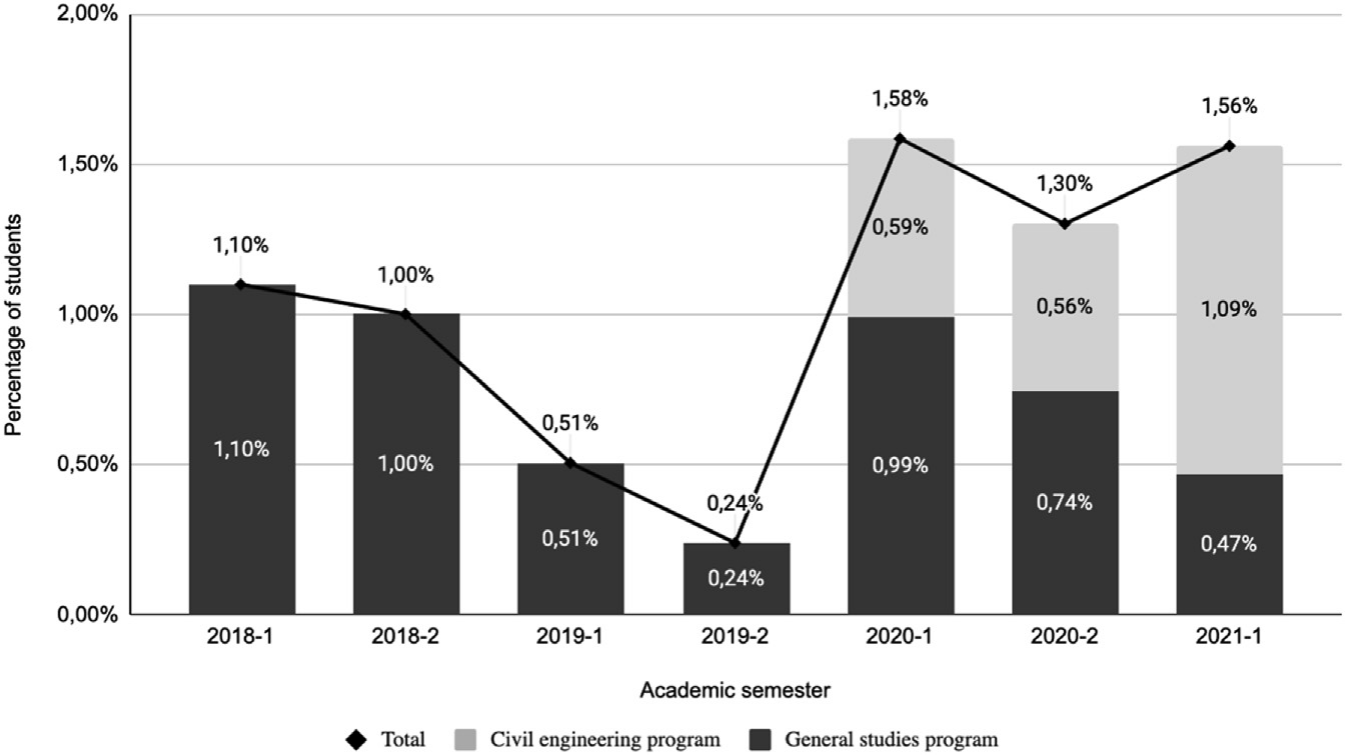
Student dropout rates within a civil engineering program, from 2018 to 2021.

Figure 1 presents the percentages that correspond to the total dropout cases compared to the total number of students enrolled in every academic semester, as reported in Table 1. Three different moments, which reveal three strong tendencies, were identified regarding the general dropout rates, as shown in Figure 1. As anticipated, these directly relate to the COVID-19 pandemic: before it, when it first burst, and during it. From 2018-1 to 2019-2, which corresponds to the first identified moment, matching when the civil engineering program was launched and started to gain recognition, the dropout rates have a downward trend, which began at 1.10% and fell up to 0.24%.

Since the decrease in dropout rates is associated with the increase in academic motivation (Rump et al., 2017), which, at the same time, is proven to grow with age (Morgan and Robinson, 2013), this trend can be explained. Thus, there is a higher probability of dropping out among the youngest students (Hovdhaugen, 2009; Meyer and Marx, 2014) compared to older students. In this context, while more time from the program’s launching passed, the population’s average age rose. Therefore, a decrease in the dropout rates can be associated with the consolidation of the undergraduate program.

Then, in 2020-1, the second moment, a sudden rise and a change to an upward trend are observed. This answers the direct impact of the outbreak of the pandemic, as reported by literature (McKinsey and Company, 2020). Since this outbreak implied the transition to online education, which is closely related to higher dropout rates than face-to-face programs (Dash et al., 2021; Grau-Valldosera and Minguillón, 2014), the results obtained can be explained.

Finally, from 2020-1 to 2021-1, the third moment corresponds to the pandemic context. The undergraduate student dropout rate has been stabilized at around 1.6%, with a variability of 0.3%. This is possibly related to dissatisfaction with the online training process (Khawar et al., 2021; Fruehwirth et al., 2021; Browning et al., 2021). Moreover, this stabilization can be explained by observing the behavior of the dropout rates in other epidemics (Chavez Villegas et al., 2021; Smith, 2021).

**Figure 2:**
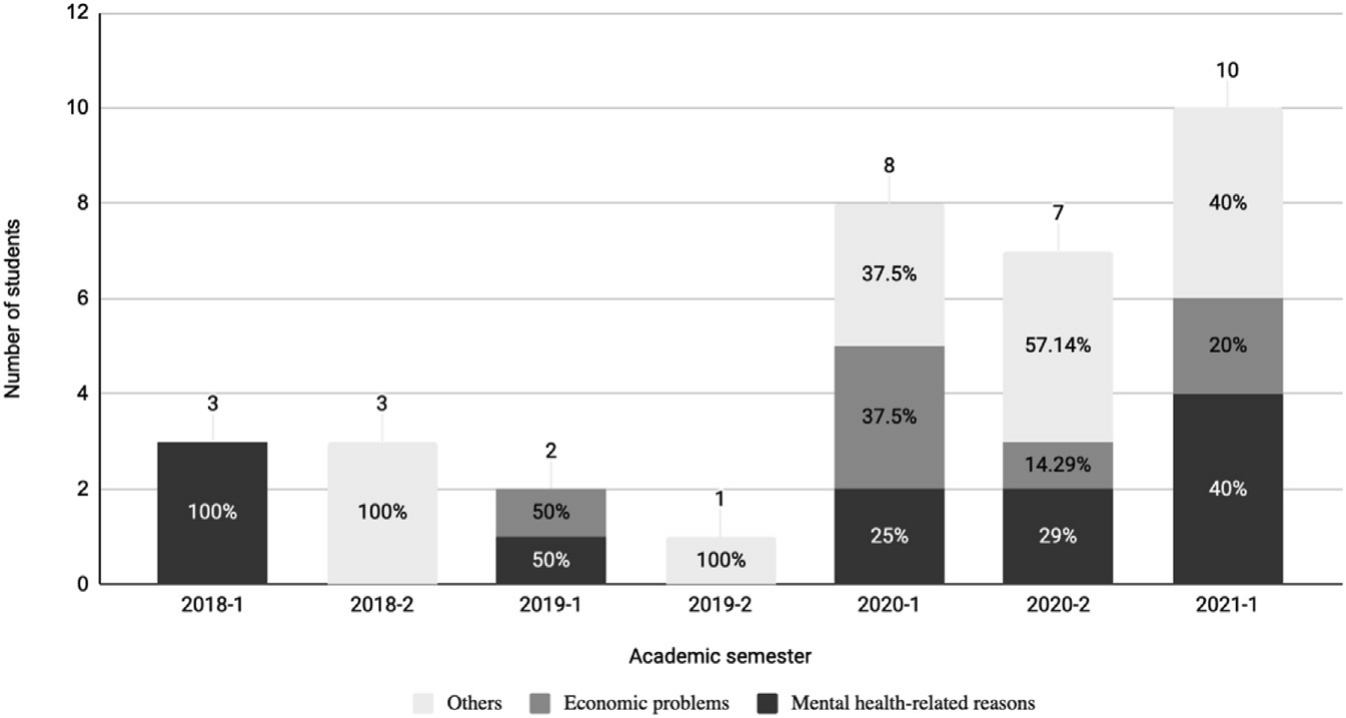
Motives for the student dropouts within a civil engineering program, from 2018 to 2021.

Figure 2 exhibits the motives for student dropouts from the total number of cases identified in each academic semester within a civil engineering program from 2018 to 2021. About these, three were considered: mental health-related reasons, economic factors, and others. Since this research focuses only on the ones related to mental health, the other ones will not be addressed. However, it must be said that, in 2020 and 2021, those “others” cases include students getting the COVID-19, as it has been previously reported (McKinsey and Company, 2020).

As Figure 2 shows, the dropout cases were isolated from 2018-1 to 2019-2, and no trend regarding the dropouts’ motives can be detected. However, from 2020-1 to 2021-1, which corresponds to the third identified moment in Figure 1, an upward trend regarding the mental health-related reasons dropouts, which go from 25% to 40%. As it is known, mental health has been a significant issue for humanity during the pandemic since it has been widely undermined, especially among undergraduate students (Rettie and Daniels, 2021; Elmer et al., 2020; Fruehwirth et al., 2021; Padrón et al., 2021).

Mental health-related reasons were the most common motives for dropout addressed by the students, as reported by similar studies (Ishii et al., 2018; NAMI, 2012). Poor mental health is associated with students’ intention to leave their studies (Chi et al., 2021). This can also clarify why the total number of dropout cases has increased from 2020-1, as Figure 2 reveals. In this line, the emotional and psychological conditions reported mainly by students with mental health problems were mixed anxiety and depressive disorder, anxiety disorder, or even depression. These conditions are analogous to what was addressed by others (Browning et al., 2021; Fruehwirth et al., 2021; Hossain et al., 2021).

Furthermore, these conditions, related to poor mental health, have a negative impact on motivation and identified regulation (Løvoll et al., 2017), which are also predictors of students’ intention to dropout (Rump et al., 2017; Vallerand et al., 1997). Therefore, it can be assumed that poor mental health and specifically anxiety and depression, can be predictors of undergraduate student dropout, and not only the intention to dropout.

**Figure 3:**
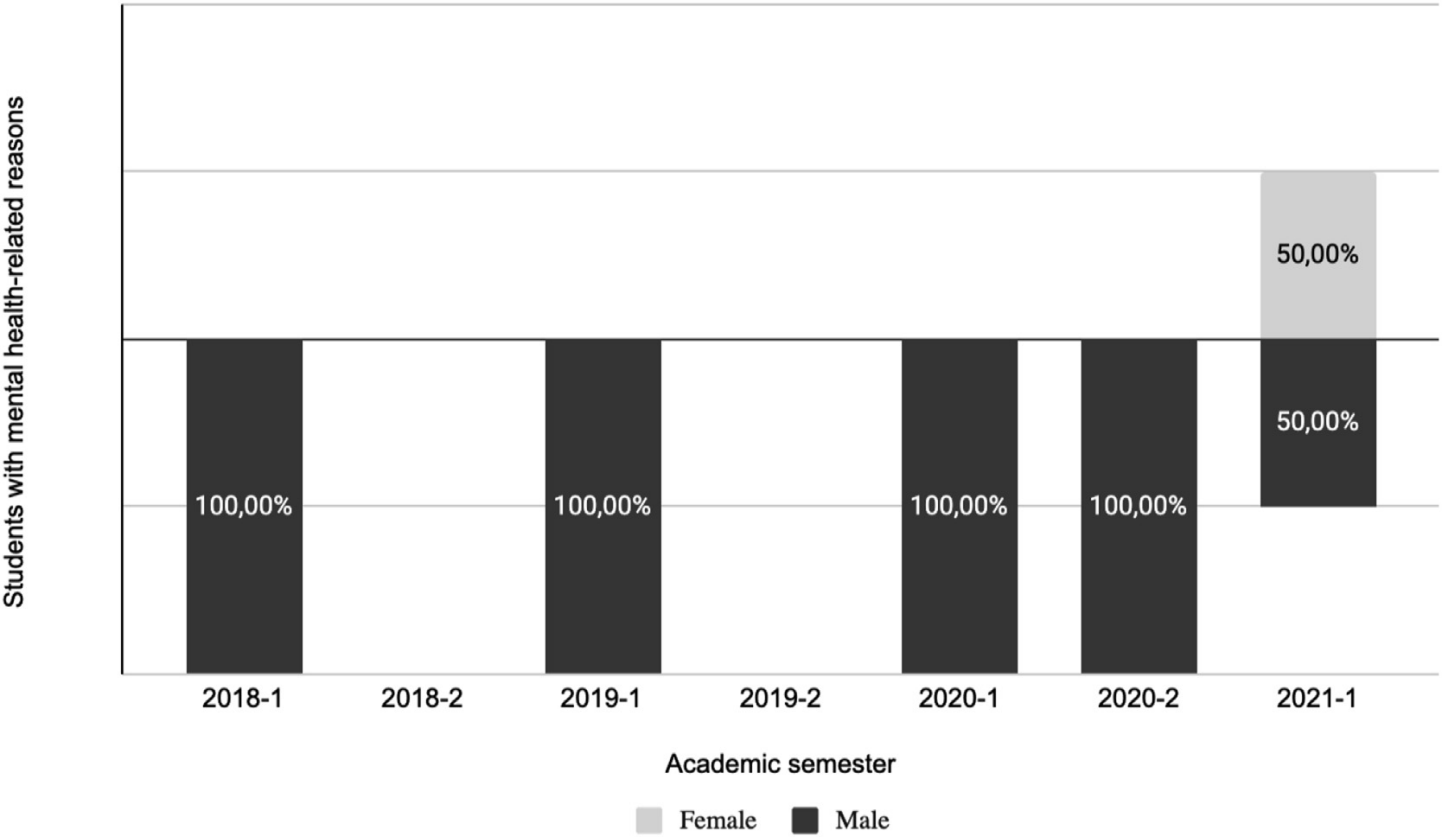
Mental health-related reasons associated with student dropouts by gender, within a civil engineering program, from 2018 to 2021.

Figure 3 shows mental health-related problems associated with student dropouts by gender, within a civil engineering program, from 2018 to 2021. The percentages addressed refer to the number of mental health-related reasons dropouts cases. Figure 3 reveals that females were most recently affected by mental health-related problems. In fact, before 2021-1, only males have been reported to dropout due to this motive, which is consistent with their higher intention to dropout in comparison to females (Hovdhaugen, 2009; Arias and Dehon, 2013). This gender difference can be explained by the resilience factor, which is more prevalent in females (Ayala and Manzano, 2018; Netuveli et al., 2008). Therefore, the pandemic has taken longer to get to them since they can overcome difficulties and adapt through adversities better than males.

Additionally, since females report higher values of autonomous motivation compared to males (Vallerand et al., 1997), it can be concluded that it is less probable to see them dropping out in comparison with males. Finally, the results are also aligned to the population's distribution since the civil engineering program has an approximate ratio of 1:4, women and men, respectively.

## 4 Conclusions

Regarding the differences in the undergraduate dropout rate of civil engineering students from a private university before and after the COVID-19 pandemic, it was concluded that this rate has increased by around 1.24%. Three different tendencies for these rates were identified, which are a direct consequence of the COVID-19 pandemic: before it, when it first burst, and during it. Along with the last tendency, the dropout rates for mental health-related reasons have increased, going from 0 to 25–40%. Before the pandemic, mental health was not a constant motive for student dropout. Therefore, it is expected that, when the pandemic is over, the dropout rates will return to their downward trend and will consolidate in about 0.25% or less.

Additionally, the gender factor should be considered to explain dropouts. No cases of female dropouts were reported before the pandemics. Since studies have identified higher resilience and motivation in them compared to males, it can be concluded that they adapt and stay focused more easily. This study has also proven that the intention of dropping out can be extended to the action.

As for the practical implications and the significance of this research in the context, it is expected that the results obtained are a step towards raising wellbeing awareness within the staff and students of the civil engineering program. Workshops will be developed to disseminate this research and, at the same time, provide professors and students with information about the importance of looking after their mental health.

Finally, authors should monitor these rates throughout and after the pandemic to detect other tendencies that allow proper action for future works. It would also be relevant to extend this study with a national or international scope to generalize. Additionally, as the COVID-19 pandemic progresses, it would be valuable to develop practical research regarding early detection of mental health-related problems within the classroom context and personalized assistance mechanisms for students to prevent them from actually dropping out.

## Declarations

### Author contribution statement

A.A. Del Savio; K. Galantini; A. Pachas: Conceived and designed the experiments; Performed the experiments; Analyzed and interpreted the data; Contributed reagents, materials, analysis tools or data; Wrote the paper.

### Funding statement

This research did not receive any specific grant from funding agencies in the public, commercial, or not-for-profit sectors.

### Data availability statement

The data that has been used is confidential.

### Declaration of interests statement

The authors declare no conflict of interest.

### Additional information

No additional information is available for this paper.
